# Expression and glucocorticoid-dependent regulation of the stress-inducible protein DRR1 in the mouse adult brain

**DOI:** 10.1007/s00429-018-1737-7

**Published:** 2018-08-18

**Authors:** Mercè Masana, Sören Westerholz, Anja Kretzschmar, Giulia Treccani, Claudia Liebl, Sara Santarelli, Carine Dournes, Maurizio Popoli, Mathias V. Schmidt, Theo Rein, Marianne B. Müller

**Affiliations:** 1 Max Planck Institute of Psychiatry, Kraepelinstr. 2-10, 80804 Munich, Germany; 2 Translational Psychiatry, Department of Psychiatry and Psychotherapy and Focus Program Translational Neuroscience (FTN), Johannes Gutenberg University Medical Center, Hanns-Dieter-Hüsch-Weg 19, 55128 Mainz, Germany; 3 Department of Biomedical Sciences, Faculty of Medicine and Health Sciences, University of Barcelona, IDIBAPS, CIBERNED, Barcelona, Spain; 4 Laboratory of Neuropsychopharmacology and Functional Neurogenomics, Dipartimento di Scienze Farmacologiche e Biomolecolari and CEND, Università di Milano, Milan, Italy; 5 Translational Neuropsychiatry Unit, Department of Clinical Medicine, Aarhus University, Risskov, Denmark; 6 Deutsches Resilienz-Zentrum, Mainz, Germany

**Keywords:** Fam107A, Tu3a, Stress, Glucocorticoids, Brain protein expression, Brain mRNA expression

## Abstract

Identifying molecular targets that are able to buffer the consequences of stress and therefore restore brain homeostasis is essential to develop treatments for stress-related disorders. Down-regulated in renal cell carcinoma 1 (DRR1) is a unique stress-induced protein in the brain and has been recently proposed to modulate stress resilience. Interestingly, DRR1 shows a prominent expression in the limbic system of the adult mouse. Here, we analyzed the neuroanatomical and cellular expression patterns of DRR1 in the adult mouse brain using in situ hybridization, immunofluorescence and Western blot. Abundant expression of DRR1 mRNA and protein was confirmed in the adult mouse brain with pronounced differences between distinct brain regions. The strongest DRR1 signal was detected in the neocortex, the CA3 region of the hippocampus, the lateral septum and the cerebellum. DRR1 was also present in circumventricular organs and its connecting regions. Additionally, DRR1 was present in non-neuronal tissues like the choroid plexus and ependyma. Within cells, DRR1 protein was distributed in a punctate pattern in several subcellular compartments including cytosol, nucleus as well as some pre- and postsynaptic specializations. Glucocorticoid receptor activation (dexamethasone 10 mg/kg s.c.) induced DRR1 expression throughout the brain, with particularly strong induction in white matter and fiber tracts and in membrane-rich structures. This specific expression pattern and stress modulation of DRR1 point to a role of DRR1 in regulating how cells sense and integrate signals from the environment and thus in restoring brain homeostasis after stressful challenges.

**Electronic supplementary material:**

The online version of this article (10.1007/s00429-018-1737-7) contains supplementary material, which is available to authorized users.

## Introduction

Stress induces a plethora of molecular changes in the brain aimed at facilitating individual adaptation to new environmental demands. However, in individuals at risk, stress can trigger deleterious consequences on neuronal function through mechanisms that are not fully understood (de Kloet et al. 2005; Popoli et al. 2012; Sousa and Almeida 2012; Duman et al. 2016). Glucocorticoid receptor (GR) activation induces gene transcription and mediates some—but not all—of the long-term effects of stress. GR-mediated mechanisms are prominent in late phase response to stress (slow stress-induced changes) and appear to be involved in homeostasis restoration and consolidation of relevant information for future use (de Kloet et al. 2005; Yau and Seckl 2012). Thus, the characterization of GR targets responsible for restoring homeostasis and attenuating the negative consequences of stress is a promising route for the development of treatments for stress-related disorders.

The stress-inducible gene down-regulated in renal cell carcinoma 1 (DRR1, also known as TU3A and Fam107A) was initially identified as a putative tumor suppressor gene in renal cell carcinoma (Yamato et al. 2000; Wang et al. 2000), then as unique gene increased in both psychiatric and neurodegenerative disorders (Shao and Vawter 2008; Li et al. 2014; Shin et al. 2016) and recently pointed as a novel molecular player promoting stress resilience (Masana et al. 2014, 2015; van der Kooij et al. 2016), supporting the involvement of DRR1 in modulating neuronal function under pathophysiological conditions. Interestingly, DRR1 is an actin-interacting protein and during recent years, neuronal actin dysfunction has been suggested as a potential shared pathological mechanism in neurodevelopmental disorders (van der Kooij et al. 2016; Yan et al. 2016).

GR activation increases DRR1 gene expression in stress-relevant brain regions such as the hypothalamic paraventricular nucleus and the hippocampal CA3 region (Liebl et al. 2009; Schmidt et al. 2011), lateral septum (Masana et al. 2014) and prefrontal cortex (Stankiewicz et al. 2014). Virus-induced overexpression of DRR1 in these brain regions improves cognitive performance (Schmidt et al. 2011) and increases social behavior (Masana et al. 2014), suggesting that DRR1 facilitate specific behaviors which might be protective against some of the deleterious consequences of stress exposure.

Interestingly, DRR1 protein is strongly conserved among species (Zhao et al. 2007; Schmidt et al. 2011) and particularly involved in the development of the nervous system (Pankratz et al. 2007; Zhao et al. 2007; Asano et al. 2010; Pollen et al. 2015). During mouse embryonic development, DRR1 is expressed in axonal projections of the central and peripheral nervous system (Asano et al. 2010). The neuroanatomical DRR1 mRNA distribution examined so far in the adult mouse brain reveals a distinct spatial DRR1 expression pattern (Schmidt et al. 2011). However, an exhaustive characterization of DRR1 expression in the adult mouse brain has not been conducted so far.

In the present study, we aimed to (1) thoroughly characterize DRR1 mRNA and protein expression pattern in the adult mouse brain and (2) to identify in which brain regions and subcellular localization DRR1 expression is modulated by glucocorticoids.

## Materials and methods

### Animals

Male C57/Bl6N mice (Charles River Laboratories) (> 12 weeks old) were used for all experiments. Animals were single housed and kept on a 12-h light/dark cycle (lights on at 7:00 AM), at room temperature of 23 ± 2 °C, with food and water provided ad libitum. All efforts were made to minimize animal suffering during the experiments. All experiments were carried out in the animal facilities of the Max Planck Institute of Psychiatry in Munich, Germany.

### Dexamethasone treatment

Dexamethasone-21-dihydrogen-phosphate disodium salt (DEX) (Fortecortin^®^-inject 100 mg (10 ml), Merck Pharma GmbH, Germany), a potent synthetic agonist of the glucocorticoid receptor, was diluted to a final concentration of 2 mg/ml with 0.9% saline and injected sub-cutaneously (s.c.) with a single dosage of 10 mg/kg body weight between 8 and 9 am (~ 130 μl). Vehicle-treated animals (Veh, control) were injected with the corresponding volume of 0.9% saline. All animals were killed 8 h post-injection under isoflurane anesthesia, trunk blood samples collected for corticosterone levels assessment using a radioimmunoassay kit (MP Biomedicals Inc; sensitivity 6.25 ng/ml) as previously described (Wagner et al. 2011), and brains processed for in situ hybridization and Western blot analysis. Three batches of animals were used. First batch (*n* = 6 vehicle; *n* = 6 DEX): one brain hemisphere was used for hippocampal total protein extraction and analysis, the other for in situ hybridization. Second batch (*n* = 6 Vehicle; *n* = 6 DEX): both hippocampi were dissected and subjected to subcellular fractionation for protein analysis (cytosol, membrane, nuclei, cytoskeleton). Third batch: 6 hippocampi pools/group (4 mice hippocampi/pool) were used for subcellular fractionation.

### In situ hybridization

In situ hybridization was performed as previously reported (Schmidt et al. 2011; Masana et al. 2014, 2015). Brains were quickly removed, frozen in isopentane and stored at − 20 °C. Sections were cut at 18 µm using a cryostat microtome, thaw-mounted on Superfrost plus slides (Menzel Gläser, Germany), and stored at − 20 °C. The antisense cRNA hybridization probe was 486 bp long, covering exons 1–4 (forward primer: GTGGAGGGAAGGAGAAGGAC; reverse primer: TCCCGGACTTTGATGAACTC) and using Basic Local Alignment Tool (BLAST, NCBI, NIH) optimized for both highly similar sequences (megablast) and more dissimilar sequences (discontiguous megablast), indicate that our riboprobe would only bind to one transcript corresponding to DRR1. The intensity of the DRR1 mRNA expression has been shown to increase in virus-mediated DRR1 overexpression (Schmidt et al. 2011; Masana et al. 2014). Sections were fixed in 4% paraformaldehyde, acetylated in 0.25% acetic anhydride and dehydrated in increasing concentrations of ethanol. Each slide was exposed to 100 µl of hybridization buffer containing 3–5 × 10^6^cpm [^35^S] labeled riboprobe, coverslipped and incubated overnight at 55 °C. The next day, sections were rinsed in 2x standard saline citrate (SSC), treated with RNAse A (20 mg/l) at 37 °C and washed in decreasing SSC concentration solutions at room temperature. Then, sections were washed in 0.1 × SSC for 1 h at 65 °C and dehydrated through increasing concentrations of ethanol. Finally, slides were exposed to Kodak Biomax MR films (Eastman Kodak Co., Rochester, NY, USA), developed and the autoradiographs digitized. For increased resolution of the neuroanatomical expression pattern of DRR1 mRNA a silver grain staining was performed. Sections were dipped in Kodak NTB emulsion (Eastman Kodak Co., Rochester, NY), exposed for 3 days at 4 °C, and then developed in Kodak D19 solution. The developed slides were lightly counterstained with cresyl violet.

### Immunofluorescence

Immunofluorescence stainings were performed as previously described (Schmidt et al. 2011; Masana et al. 2014). Animals were deeply anesthetized with ketamin/rompun and perfused intracardially with 4% paraformaldehyde; brains were removed, post-fixed overnight in 4% paraformaldehyde following overnight incubation in 20% sucrose solution at 4 °C, frozen in isopentane at − 40 °C. All brain tissue was subsequently stored at − 20/− 80 °C until use. Free-floating sections were cut at 30 µm using a cryostat microtome, thaw-mounted on Superfrost slides (Menzel Gläser, Germany) and stored at − 20 °C until immunofluorescence staining was performed. Phosphate buffer 0.1 M (PB) was used for all washing steps, and all reagents were diluted in PB. Sections were washed in 0.3% Triton-X100, blocked for 1 h in 5% normal donkey serum (Abcam) in 0.3% Triton-X100, and incubated with the primary antibodies overnight at 4 °C. Sections were washed and incubated for 2 h with Alexa Fluor-conjugated secondary antibodies (1:500) at room temperature, and washed again. Then, rinsed in distilled water, mounted on superfrost plus slides, and covered with Vectashield mounting medium (Vector Laboratories, Burlingame, USA) containing DAPI. The rabbit polyclonal antibody against DRR1 was produced by Biogenes (Berlin, Germany) using recombinant DRR1 in rats and affinity-purified (with the same antigen) (Schmidt et al. 2011) and it has been used for Western blotting and immunostaining before (Masana et al. 2014; Schmidt et al. 2011). While there is no DRR1-knock-out available, the specificity of the antibody had been assessed as follows: (1) In Western blot analyses, the antibody recognizes recombinant DRR1, as well as DRR1 in extracts from cells transfected to express DRR1 and from brain tissue expressing DRR1 (Schmidt et al. 2011). (2) The intensity of the DRR1 protein expression by immunofluorescence increases in virus-mediated overexpression (Schmidt et al. 2011; Masana et al. 2014). Also mouse anti-neuronal nuclei (NeuN) (1:500; Millipore); goat anti-synaptophysin (1:250; SantaCruz); goat anti-PSD-95 (1:500; Abcam) and the appropriate secondary antibodies linked to Alexa Fluor 488/555/647 (Invitrogen) were used.

### Image acquisition and analysis

Silver grain stainings of in situ hybridization were examined using an Axioskop2 microscope and photomicrographs were taken with Axiovision software (Zeiss, Jena, Germany). Images of immunofluorescence stainings were obtained with a laser-scanning confocal microscope (IX81-FV1000, Olympus, Tokyo, Japan) using Olympus FV10-ASW 2.0 software. All images were processed with the FIJI software (Schindelin et al. 2012). Representative images were adjusted for brightness and contrast. Overview figures were created from 16 to 30 single images using the MosaicJ plugin (Thévenaz and Unser 2007). The different anatomical brain regions were identified with the mouse brain atlas (Paxinos and Franklin 2008). Signal intensities were rated into one of the following categories: − not detectable, + weak signal, ++ moderate signal and +++ strong signal, according to the grey-intensity levels in the autoradiograph and also the expression observed in the high-resolution photomicrographs of [^35^S]-labeled DRR1 mRNA. Densitometric quantification of autoradiographs of [^35^S]-labeled mRNA was done using the Fiji software and grey values were expressed as arbitrary units (a. u.). Four different brain slices/ animal were measured. Background signal was subtracted outside the tissue section.

#### Western blot

For total protein detection, hippocampi were dissected and frozen on dry ice, homogenized in 100 μl ice-cold lysis buffer (50 mM Tris/HCl, 250 mM sucrose, 1 mM EDTA, 1:100 protease inhibitor cocktail P2714 (Sigma Aldrich), pH adjusted to 7.5), centrifuged 10 min at 8000 rpm, the supernatant was centrifuged again 10 min at 7000 rpm, at 4 °C. Cytosol (F1), membranes (F2), nuclear (F3) and cytoskeletal (F4) fractions were separated using a commercially available kit (ProteoExtract^®^ Subcellular Proteome Extraction Kit, Calbiochem, 539790). Protein concentrations were determined using a detergent compatible protein assay kit (Bio-Rad, Hercules, CA, USA). Samples containing 40 µg of protein were resolved by 15% sodium dodecyl sulphate–polyacrylamide gels, and transferred onto nitrocellulose membranes (GE Healthcare).

A second subcellular separation was done according to the protocol described in Gardoni et al. (2006) and Treccani et al. (2014). Each pool of tissue was homogenized in 0.32 M ice-cold sucrose containing (in mM): 1 HEPES, 1 MgCl_2_, 1 EDTA, 1 NaHCO_3_, and 0.1 PMSF, at pH 7.4, with a complete set of protease inhibitors (Complete; Roche Diagnostics, Basel, Switzerland) and phosphatases inhibitors (Sigma, St. Louis, MO, USA). The hippocampi homogenate was centrifuged 10 min at 1000*g*. The obtained supernatant (S1) was centrifuged 15 min at 3000*g*. The cytosol fraction was obtained (S2 fraction) and the pellet (P2 fraction, crude membrane fraction) was resuspended in 1 mM HEPES plus CompleteTM in a glass–glass potter and centrifuged 1 h at 100,000*g*. The pellet (P3 fraction) was resuspended in buffer containing 75 mM KCl and 1% Triton X-100 and centrifuged 1 h at 100,000*g*. The supernatant (S4 fraction, Triton X-100-soluble fraction (TSF)) was obtained and the final pellet (P4 fraction, TIF) was homogenized in a glass–glass potter in 20 mM HEPES, an equal volume of glycerol was added and stored at − 80 °C until processing.

Membranes were labeled with rabbit anti-DRR1 (1:2000, BioGenes), goat anti-actin (1:2000, Santa Cruz Biotechnology), rat anti-tubulin (1:5000, Abcam) antibodies overnight at 4 °C. Following incubation with the corresponding horseradish peroxidase-conjugated (1:2000, DAKO) secondary antibodies for 3 h. Bands were visualized using an enhanced chemiluminescence system (Millipore), detected using the Chemidoc system (BioRad) and quantified by densitometry (Image Lab Software, Bio-Rad). Duplicates of each sample were loaded and measured independently.

### Statistical analysis

The commercially available program GraphPad Prism 4 was used for statistical analysis. Simple group comparisons were performed using the two-tailed unpaired *t* test. If more than two groups were compared, an ANOVA was performed followed by Bonferroni post hoc analysis. The level of significance was set at *p* < 0.05. All data are presented as mean + SEM.

## Results

### DRR1 mRNA expression in the adult mouse brain

**Fig. 1:**
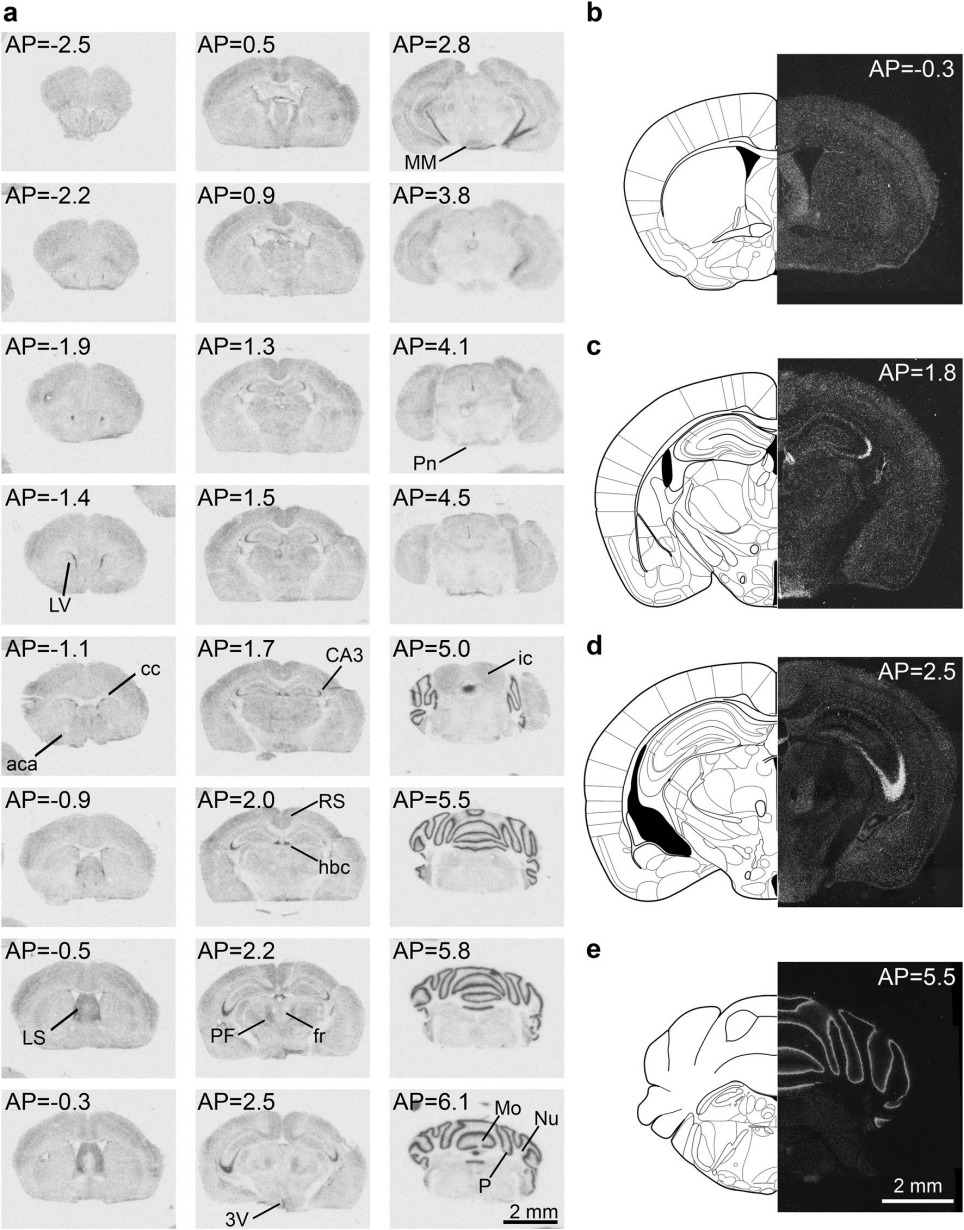
DRR1 mRNA showed distinct expression throughout the adult mouse brain. **a** Representative autoradiograms of [^35^S]-labeled DRR1 coronal brain sections from the same mouse at different anterioposterior (AP) location in relation to bregma. **b**–**e** High-resolution photomicrographs showing the expression pattern of DRR1 mRNA revealed by silver grain staining under dark-field illumination. Pictures show mosaics of several photomicrographs at **b** AP: − 0.3; **c** AP: 1.8; **d** 2.5 and **e** 5.5 mm from bregma (Paxinos and Franklin 2008). *aca* Anterior commissure, *cc* corpus callosum, *CA3* cornu ammonis 3, *fr* fasciculus retroflexus, *hbc* habenular commissure, *ic* inferior colliculus, *LS* lateral septum, *LV* lateral ventricle, *MM* mammillary nucleus, *Mo* molecular layer of the cerebellum, *Nu* nuclear layer of the cerebellum, *PF* parafascicular thalamic nucleus, *Pn* pontine nuclei, *P* Purkinje cell layer of the cerebellum, *RS* retrosplenial cortex, *3V* third ventricle

DRR1 mRNA is widely expressed in the whole adult mouse brain (Fig. 1; S1; S2; Table 1) with a distinct neuroanatomical expression intensity profile. From anterior to posterior relative to bregma, the strongest DRR1 mRNA expression was detected in the posterior part of the lateral septum; the subfornical organ; the cell bodies of the pyramidal CA3 region of the hippocampus, specifically the CA3a and initial part of CA3b subfield; and cells ensheathing the stalk of the habenular commissure and the Purkinje cell layer of the cerebellum.

**Table 1:** DRR1 expression in the mouse brain

Brain structure	mRNA levels	Protein levels
Cortex		
Layer I	++	++
Layer II	++	++
Layer III	++	++
Layer IV	++	+++
Layer V	−/+	++
Layer VI	++	++
Hippocampal region		
CA3	+++	++
CA2	+	+
CA1	+	+
Granular layer of the dentate gyrus	+	+
Polymorphic layer of the dentate gyrus	+	+
Molecular layer of the dentate gyrus	+	++
Stratum lacunosum-moleculare	+	++
Stratum radiatum	−	+
Septal region		
Lateral septal nucleus	+++	++
Medial septal nucleus	++	++
Septohippocampal nucleus	++	++
Septofimbrial nucleus	+	+
Bed nucleus of anterior commissure	n/a	++
Median preoptic nucleus	n/a	++
Basal ganglia and related areas		
Caudate putamen	++	++
Globus pallidus	+	++
Bed nucleus of the stria terminalis	+	++
Substantia nigra	−	+
Subthalamic nucleus	+	++
Thalamus		
Habenular nuclei	+	++
Habenular commissure	+++	+++
Reuniens thalamic nucleus	+	++
Parafascicular thalamic nucleus	++	++
Zona incerta	++	++
Ventral pallidum	+	++
Posterior thalamic nuclear group	+	++
Dorsal/ventral lateral geniculate nucleus	+	++
Bed nucleus of the stria terminalis	+	++
Hypothalamus		
Paraventricular hypothalamic nucleus	+	+
Peri-PVN region	+	+
Ventromedial hypothalamic nucleus	++	+
Lateral mammillary nucleus	+++	+++
Medial mammillary nucleus	+++	+++
Supraoptic nucleus	n/a	++
Arcuate hypothalamic nucleus	++	++
Suprachiasmatic nucleus	++	++
Amygdaloid complex		
Basolateral amygdaloid nucleus	++	++
Basomedial amygdaloid nucleus	++	++
Central amygdaloid nucleus	+	++
Circumventricular organs		
Subfornical organ	+++	+++
Subcommissural organ	−	++
Organum vasculosum lamina terminalis	n/a	++
Median eminence	−	+
Pituitary	n/a	n/a
Area postrema	n/a	++
Cerebellum		
Purkinje cell layer	+++	+++
Molecular layer	++	+++
Granular layer	+	+
White matter structures		
Corpus callosum	+	+
Anterior commissure	−	+
Fornix	−	+
Optic tract	−	++
Ventral hippocampal commissure	−	+
Cerebral peduncle	−	+
Non-neuronal tissue		
Choroid plexus	++	++
Ventricular Ependymal cells	++	++

Relative expression levels of DRR1 mRNA and protein in adult mouse brain is expressed in the following four categories: − not detectable, + weak signal, ++ moderate signal and +++ strong signal, mRNA expression levels was classified into four categories according to intensity levels of mRNA expression in autoradiographs and photomicrographs (Figs. 1, S1, S2). Only regions that could be clearly assigned with regard to DRR1 mRNA or protein expression were included

Moderate DRR1 gene expression was visible, but not uniformly distributed, throughout the cortex. Especially high expression was detected in the primary somatosensory cortex and the retrosplenial cortex (both granular and agranular part). Interestingly, the cortical layer V (the inner pyramidal layer) displayed lower DRR1 mRNA expression compared to the other cortical layers. Moderate DRR1 expression was also found in the lateral part of the caudate putamen, the amygdala, the mammillary nuclei and several thalamic (ventromedial hypothalamic nucleus and the parafascicular thalamic nucleus) and hypothalamic nuclei.

Only very weak DRR1 mRNA expression was visible in the *mesencephalon* and the *rhombencephalic* medulla oblongata and pons.

Whereas weak to strong DRR1 expression was found in neuronal tissue throughout the brain, DRR1 mRNA expression was less observed in white matter structures like the anterior commissure, the corpus callosum, the cerebral peduncle and the optic tract. We can observe a punctate pattern in the high-resolution silver grain-stained DRR1 mRNA images; however, intensity of the signal provided by the autoradiograph is almost undetectable. Interestingly, the expression of DRR1 mRNA was also detected in the choroid plexus and in ependymal cells of the brain ventricles, similar to Fam107B (Masana et al. 2015).

### Neuroanatomical distribution of DRR1 protein

**Fig. 2:**
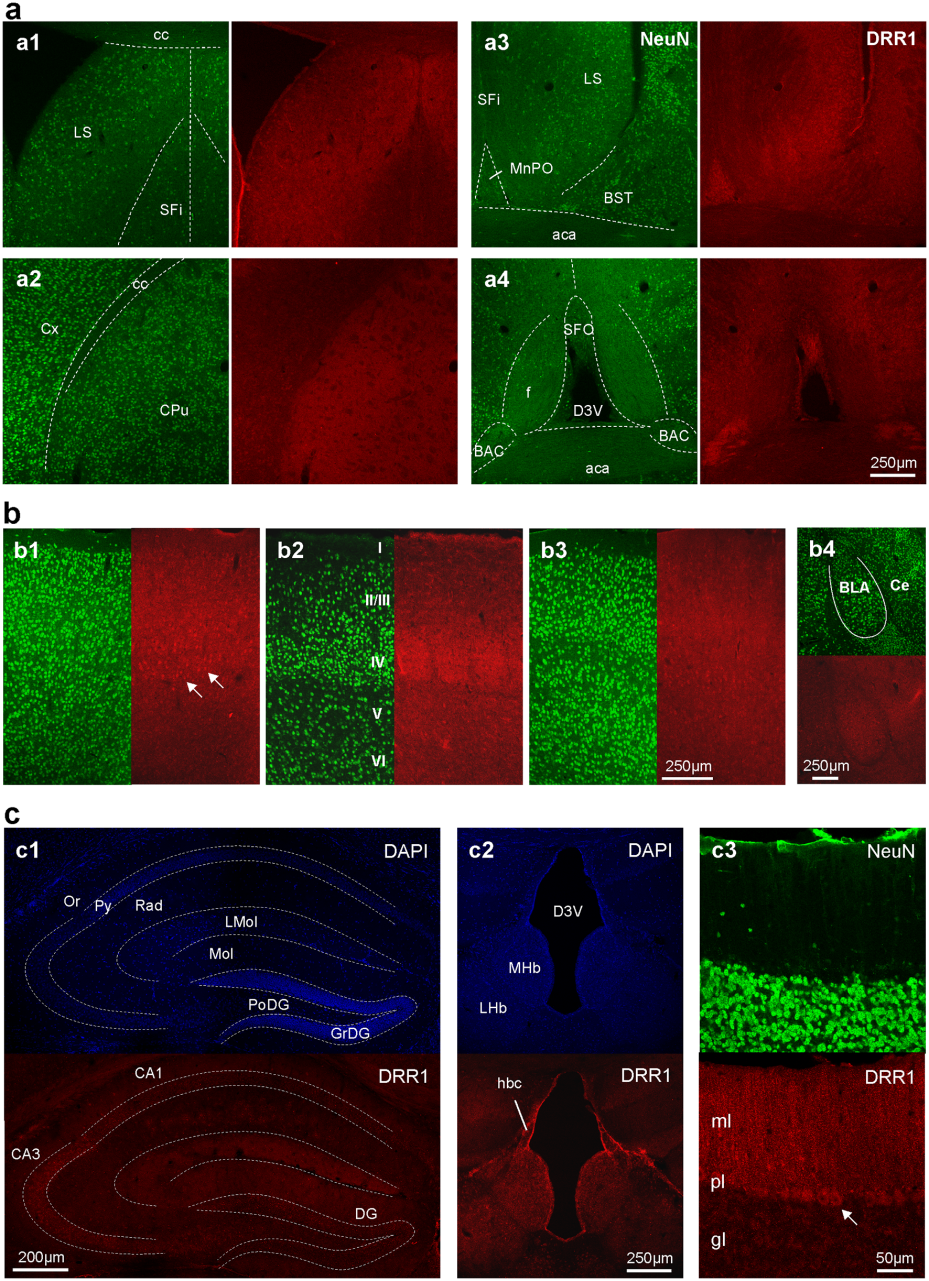
DRR1 protein is expressed in specific regions throughout the entire adult mouse brain. Representative confocal images show immunofluorescence of DRR1 (red) and NeuN (green) or DAPI (blue) from **a** the striatal and septal region (0.2 to − 0.1 mm from bregma), **b** cerebral cortex and amygdala region (− 0.7 mm from bregma) and **c** hippocampal and cerebellar regions (− 1.8 and − 5.5 mm from bregma, respectively). Specifically, expression of DRR1 was found in the lateral septum (**a1, a3**), striatum (**a2**), median preoptic nucleus (**a3**), bed nucleus of the anterior commissure and subfornical organ (**a4**), primary motor cortex (**b1**), barrel field (**b2**), secondary somatosensory cortex (**b3**), amygdala (**b4**), hippocampus (**c1**), habenular commissure (**c2**) and Purkinje cell layer and molecular layer of the cerebellum (**c3**). *aca* Anterior commissure anterior part, *BAC* bed nucleus of the anterior commissure, *BLA* basolateral amygdaloid nucleus, *BST* bed nucleus of the stria terminalis, *CA1* cornu ammonis 1, *CA3* cornu ammonis 3, *cc* corpus callosum, *Ce* central amygdaloid nucleus, *CPu* caudate putamen, *Cx* cortex, *D3V* dorsal 3rd ventricle, *DG* dentate gyrus, *f* fornix, *gl* granular layer of the cerebellum, *GrDG* granular layer of the DG, *LMol* lacunosum moleculare layer, *hbc* habenular commissure, *LS* lateral septal nucleus, *MHb* medial habenular nucleus, *LHb* lateral habenular nucleus, *MnPO* median preoptic nucleus, *ml* molecular layer of the cerebellum, *Mol* molecular layer, *Or* oriens layer, *PoDG* polymorph layer of the dentate gyrus, *pl* Purkinje cell layer of the cerebellum, Py pyramidal cell layer, *Rad* stratum radiatum, *SFi* septofimbrial nucleus, *SFO* subfornical organ

**Fig. 3:**
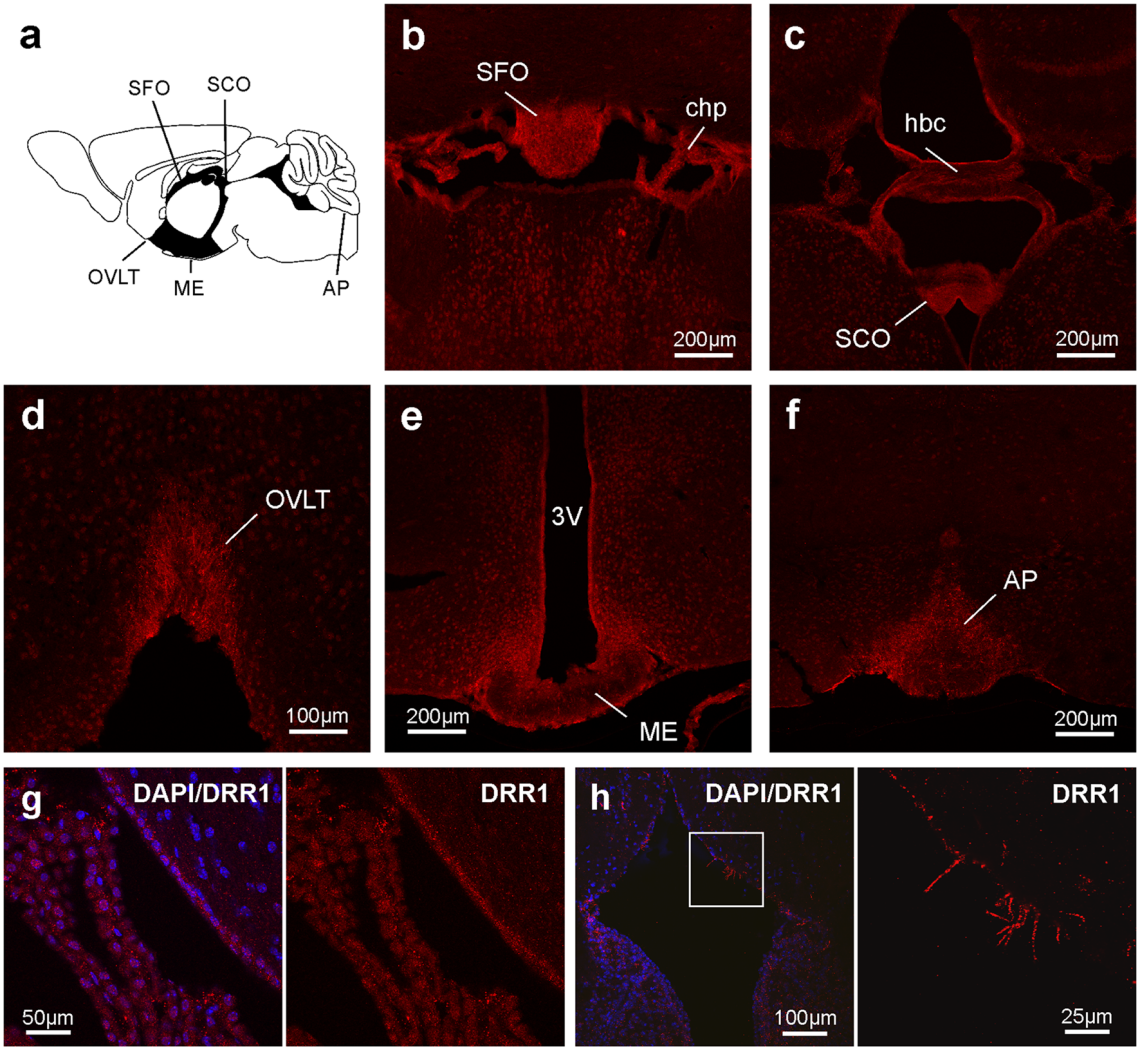
DRR1 protein is expressed in most of the circumventricular organs. **a** Schematic overview of the location of the different circumventricular organs in the mouse brain. Representative confocal images show immunofluorescence of DRR1 (red) and DAPI (blue) from **b** the subfornical organ (0.8 mm posterior to bregma), **c** the subcommissural organ (2.5 mm posterior to bregma), **d** the vascular organ of the lamina terminalis (0.50 mm anterior to bregma), **f** the area postrema, (1.7 mm posterior to bregma) **e** but not the median eminence. DRR1 protein can also be found in non-neuronal cells of **g** the choroid plexus and the ventricle ependyma. **h** In some rare cases, DRR1 was found in cilia of ependymal cells. *3V* 3rd ventricle, *AP* area postrema, *chp* choroid plexus, *hbc* habenular commissure, *ME* median eminence, *OVLT* vascular organ of the lamina terminalis, *SCO* subcommissural organ, *SFO* subfornical organ

The neuroanatomical distribution of DRR1 protein was assessed by immunofluorescence. DRR1 protein could be found throughout all the adult mouse brain, with high expression in specific regions (Table 1; Figs. 2, 3).

The DRR1 protein abundance in the septum nicely followed the pattern of mRNA expression with high DRR1 protein levels in the lateral septum. Moreover, also the caudate putamen, lateral globus pallidus, ventral pallidum as well as the nearby bed nucleus of the anterior commissure and the bed nucleus of the stria terminalis were DRR1 positive (Fig. 2a).

Within the cortex, DRR1 was found throughout all regions and layers of the cortex (Fig. 2b). Immunofluorescence outlines neuronal cell bodies of cortical neurons (arrows in Fig. 2b1) but was also located to the surrounding neuropil. Particularly high density of DRR1 signal could be observed in layer IV of the barrel field, where it appears in barrel like structures (Fig. 2b2). Whereas the mRNA signal was clearly reduced within layer V of the cortex, DRR1 protein is not. Comparable levels of DRR1 protein were also found in the amygdala (Fig. 2b4).

Moreover, strong DRR1 protein expression was present in the hippocampus and the adjacent habenular commissure (Fig. 2c1, c2). High protein levels could be found prominently in CA3 cell bodies but also in the dentate gyrus and the molecular layers. Additionally, cells covering the stalk of the habenular commissure were positive for both DRR1 mRNA and protein (Table 1; Fig. 2c2).

The cerebellum was one of the brain regions with the highest DRR1 protein level. DRR1 protein signal was strong in the Purkinje layer (cell bodies) and molecular layer of the cerebellum (Fig. 2c3). Because very strong mRNA expression of DRR1 was only visible in the Purkinje cell layer, it is likely that most of the DRR1 protein in the molecular layer belongs to Purkinje cell dendrites.

The distribution of DRR1 protein in the thalamus and hypothalamus is summarized in Table 1. Several nuclei showed moderate DRR1 protein levels as indicated. High DRR1 abundance could be observed in the mammillary nuclei while the paraventricular nucleus of the hypothalamus showed only basal levels of DRR1.

Interestingly, most of the circumventricular organs (CVOs) were strongly labeled by DRR1 immunofluorescence (Fig. 3). High levels of DRR1 could be observed in the vascular organ of the lamina terminalis, the area postrema, the subfornical organ and the subcommissural organ, but not the median eminence. A broader definition of CVOs includes also the choroid plexus, which showed prominent DRR1 mRNA and protein (Table 1; Fig. 3g). Also nuclei known to be highly interconnected with the CVOs, the supraoptic nucleus (data not shown) and the median preoptic nucleus (Fig. 2a3), do express DRR1.

DRR1 was prominently but not exclusively located to neurons, as it is found in virtually all neurons but also in non-neuronal tissue like in the cells of the choroid plexus and the ependyma (Fig. 3g, h).

### Cellular localization of DRR1 protein

DRR1 protein appeared in a punctate pattern independent of the neuroanatomical region and the intracellular localization (Figs. 2, 3). DRR1 was found in the neuropil as well as in the neuronal somata including the nuclei, where it shows denser signal. DRR1 was also located to cellular specializations as it was found in cilia of ependymal cells (Fig. 3h).

Furthermore DRR1 might be located to both presynaptic and postsynaptic structures (Figure S1). DRR1 co-localize with some of the synapsin and PSD-95 labeled structures. Thus, DRR1 appears to be a putative but not integral part of synaptic structures.

### Glucocorticoids increase DRR1 mRNA in grey and white matter of the adult mouse brain

**Fig. 4:**
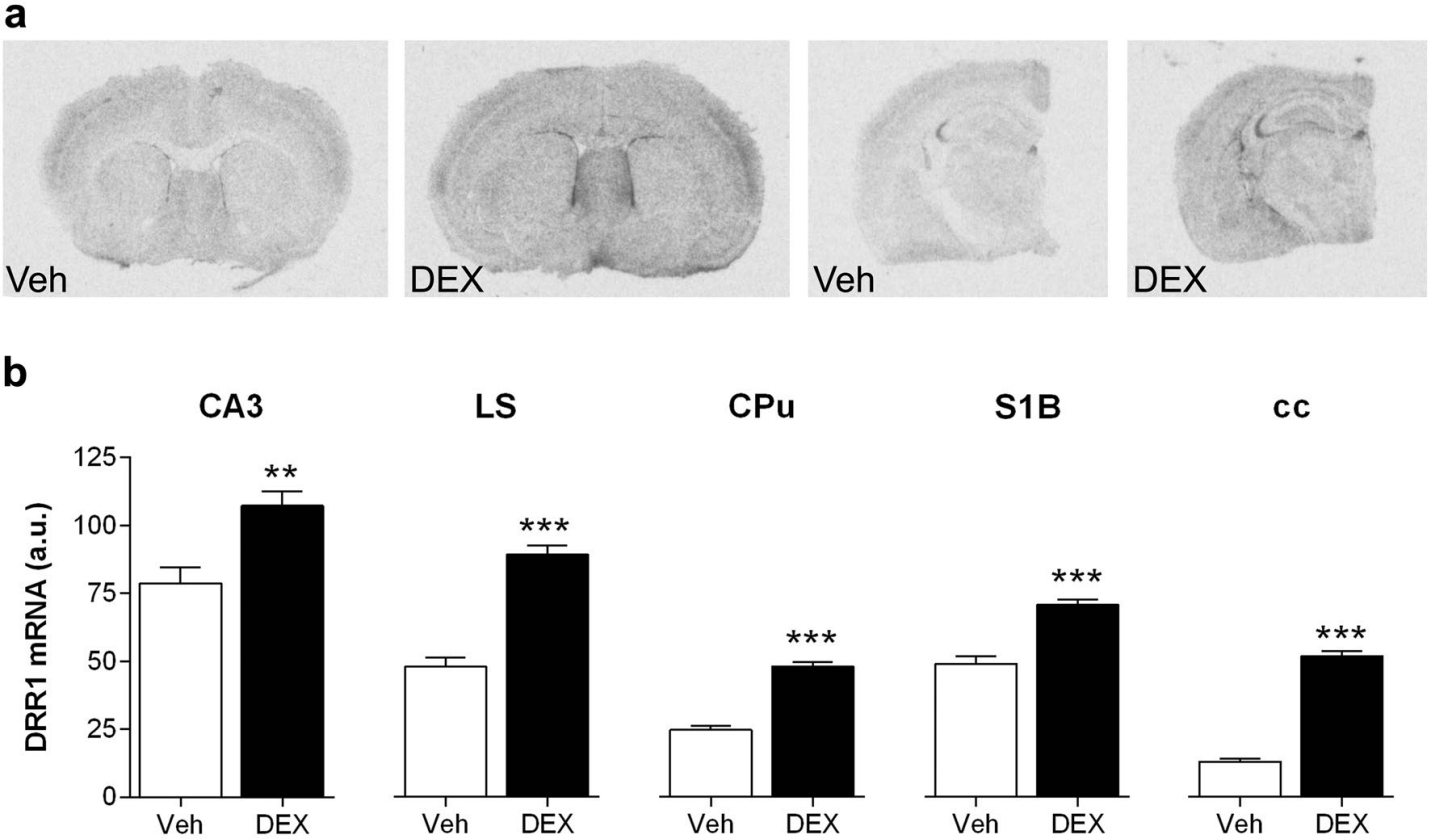
The glucocorticoid-agonist dexamethasone increased DRR1 mRNA expression in grey matter and fiber tracts. **a** Representative in situ hybridization autoradiographs of [^35^S]-labeled DRR1 mRNA of mice injected with dexamethasone (DEX) (10 mg/Kg s.c.) or vehicle (Veh). **b** Densitometric quantification of dexamethasone-induced DRR1 mRNA expression compared to vehicle. Bars show mean ± SEM of grey values in arbitrary units (a.u.). **p < 0.001 and ***p < 0.0001 vs vehicle (*t* test). *CA3* cornu ammonis 3, *cc* corpus callosum, *CPu* caudate putamen, *LS* lateral septal nucleus, *S1B* primary somatosensory cortex-Barrel field

The GR agonist dexamethasone (10 mg/Kg s.c.) increased DRR1 mRNA throughout the whole adult mouse brain, as shown in the representative autoradiographs of [^35^S]-labeled DRR1 mRNA (Fig. 4a) and densitometric analysis (Fig. 4b).

Under basal conditions, DRR1 mRNA showed maximum expression (in arbitrary units, a.u.) in the hippocampal CA3 region (79 ± 6), followed by the lateral septum (48 ± 3) and the primary somatosensorial cortex-Barrel field (49 ± 6), motor cortex (40 ± 5), striatum (25 ± 2) and with low (almost undetectable) expression in white matter fiber tracts such as the anterior commissure (12 ± 3) and corpus callosum (12 ± 3).

After glucocorticoid receptor activation, DRR1 mRNA levels increased significantly (Student’s t test) in all DRR1-rich regions but also in white matter regions. In detail, dexamethasone treatment increased mRNA expression in grey matter approximately 136% in the hippocampal CA3 region, 186% in the lateral septum, 145% in the primary somatosensorial cortex-Barrel field, 152% in the motor cortex and 195% in the striatum; while in fiber tracts increased 436% in the anterior commissure and 401% in the corpus callosum, reaching similar levels to neighboring areas such as striatum and cortex. Within the hippocampus, we could observe qualitatively increases in all CA3, DG and CA1 pyramidal neurons, and also in the str. Lac/mol and Str. Moleculare, and hilus, but this increase was less evident in the str. Radiatum. Within the cortex, DRR1 mRNA expression increased in all areas, with the somatosensory barrel field area showing the maximum expression. Moreover, the differential expression intensity pattern through the different cortical layers was maintained after dexamethasone treatment, with higher expression in layer IV and low expression in layer V.

Interestingly, DRR1 was also strongly increased in the epithelium surrounding hippocampus, around ventricles and in the choroid plexus.

### Glucocorticoids increase DRR1 in the membrane fraction

**Fig. 5:**
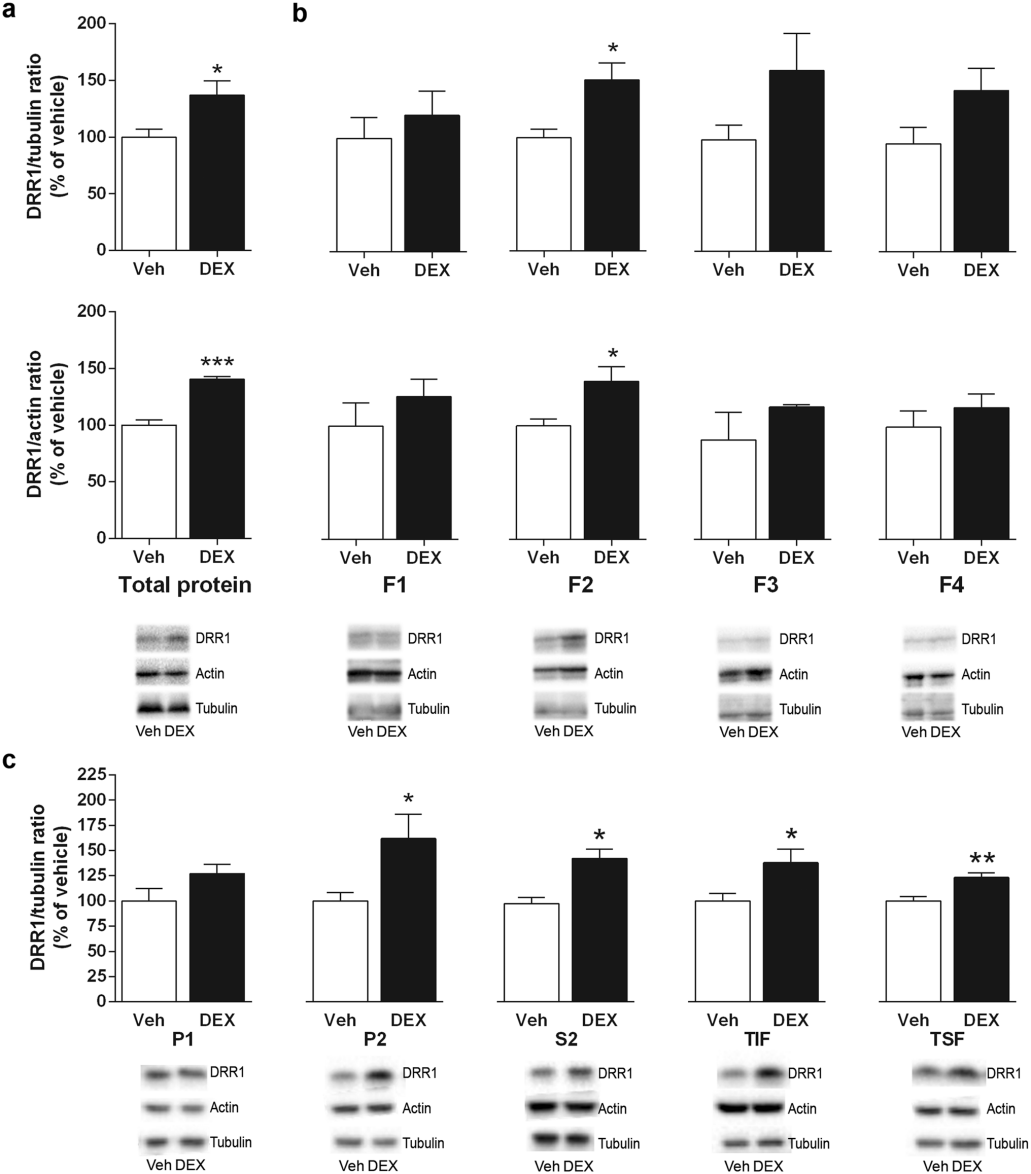
Dexamethasone (10 mg/Kg s.c.) mostly increases DRR1 protein expression close to the membrane. DRR1 protein levels were measured in the mouse hippocampus by Western Blot. **a** Total DRR1 protein levels; **b** DRR1 protein levels in the cytosol (F1), membranes (F2), nuclear (F3) and cytoskeletal (F4) subcellular fractions; and **c** DRR1 protein levels in nuclei-associated membranes (P1), crude membrane fraction (P2), cytosol (S2), Triton-insoluble fraction (TIF) and Triton-soluble fraction (TSF). Actin and tubulin were used as loading control. Bars show mean ± SEM ratios of DRR1/tubulin and DRR1/actin grey levels. *p < 0.05, **p < 0.001 and ***p < 0.0001 vs vehicle (*t* test)

DRR1 protein levels were increased by dexamethasone (10 mg/kg s.c.) treatment compared to vehicle in the mouse hippocampus, and at greater extent in membrane-rich fractions. Dexamethasone increased total protein expression of DRR1 in the hippocampus (Fig. 5a) (*p* < 0.0001 vs actin; *p* < 0.05 vs tubulin) but not in the cerebellum (data not shown) when compared to vehicle-treated mice (*n* = 6 mice/group).

Subcellular fractioning of hippocampal dissected tissue showed that DRR1 is expressed ubiquitously in the cell and dexamethasone increased DRR1 protein expression significantly in the membrane (F2) (*p* < 0.05), but not cytosol (F1), nuclei (F3) or cytoskeleton fractions (F4) (Fig. 5b) compared to vehicle-treated mice (*n* = 6 mice/group). Second subcellular fractioning showed that dexamethasone increased DRR1 in the crude membrane fraction (P2) (*p* < 0.05), cytosol (S2) (*p* < 0.05), postsynaptic membrane fraction (Triton-insoluble fraction, TIF) (*p* < 0.05) and Triton-soluble fraction (TSF) (*p* < 0.05) [when normalized to tubulin; all of them when normalized to actin (not shown)] (Fig. 5c). In all experiments corticosterone levels were measured and were significantly reduced in dexamethasone-treated mice compared to vehicle: 46.8 ± 10.6 and 0.7 ± 0.3 ng/ml for the total protein/subcellular fraction experiment in Veh and DEX-treated mice, respectively (*n* = 11–12 mice/group); and 66.6 ± 10.6 and 1.2 ± 1.3 ng/ml for the alternative subfraction procedure in Veh and DEX-treated mice, respectively (*n* = 16 mice/group). Corticosterone levels were measured and were significantly reduced in dexamethasone-treated mice compared to vehicle (Figure S4).

## Discussion

The characterization of molecular targets able to modulate stress resilience is fundamental in the search for treatments of stress-related disorders. The stress-inducible protein DRR1 was recently proposed as a novel molecular player able to modulate and attenuate the aversive consequences of stress (Masana et al. 2014; van der Kooij et al. 2016). In the present study, we provide a comprehensive description of the neuroanatomical expression of DRR1 in the adult mouse brain and its regulation by glucocorticoids. Our results indicate that DRR1 is ubiquitously expressed in the cell and specifically expressed in distinct brain regions under basal conditions. In addition, glucocorticoids increase DRR1 expression in membrane-rich structures in the cell and throughout the whole brain, showing greater increase in white matter tracts, where it is not detected under baseline conditions.

DRR1 is expressed in radial glia during development (Pollen et al. 2015), and later in neurons, astrocytes and oligodendrocytes (Cahoy et al. 2008; Masana et al. 2014; Hochgerner et al. 2018), with particularly strong upregulation and most highly expressed in late developing astrocytes (Cahoy et al. 2008). Here, we further extend the characterization of expression of DRR1 to non-neuronal tissue such as the choroid plexus and ependyma. These data indicate that glucocorticoids could modulate the physiology of both neuronal and non-neuronal brain cells through the modulation of DRR1 expression, probably by modulating actin reorganization and dynamics using similar mechanisms (Le et al. 2010; Schmidt et al. 2011).

In addition, DRR1 is ubiquitously expressed throughout the cell. This is shown by the expression of DRR1 in all the different subcellular fractions and the pattern of expression detected by immunohistochemistry, where the punctate DRR1 pattern can be found inside and around the nucleus and also the neuropil, as previously described for the lateral septum (Masana et al. 2014). This expression pattern is confirmed in other brain regions, and is especially clear in the cerebellum, where DRR1 is strongly expressed in the soma and dendrites of Purkinje cells. The presence of DRR1 protein in regions where mRNA expression is lacking is suggestive of DRR1 expression in distant axons and/or dendrites and is consistent with the expression of DRR1 in axonal projections found in mouse development (Asano et al. 2010). Another example would be layer V of the cortex, where nearly no mRNA signal was observed under both basal and dexamethasone stimulation, but showed protein expression. These data, together with the expression of DRR1 protein in membrane-rich structures and in the synapse, suggests that DRR1 is expressed in dendrites and/or axons, In addition, DRR1 is expressed in TIF and TSF fractions and partially co-localizes with synapsin and PSD95, indicating that DRR1 is a putative but not integral part of synaptic structures.

In the brain, DRR1 is expressed in CVOs, suggesting its potential role in the regulation of brain homeostasis (Denton et al. 1996). Interestingly, CVOs lack the blood–brain barrier and thus circulating blood can penetrate directly to the brain through the fenestrated vessels (Sisó et al. 2010). Moreover, the presence of DRR1 in cilia from epithelial cells further suggests that DRR1 containing structures could be relevant for sensory information from the cerebrospinal fluid (Singla and Reiter 2006) through changes in cytoskeletal structure and/or dynamics. Interestingly, cilia and dendrites show structural similarities (Nechipurenko et al. 2013), suggesting similar functions in neurons. In this line, DRR1 is expressed strongly in layer IV of the somatosensory barrel field, whose barrel neurons are the major target for somatosensory inputs from the thalamus (Schubert et al. 2003). The lateral septum has also a key role in integrating information from affective brain regions, especially from hippocampus and hypothalamus among others (Sheehan et al. 2004). Also, the hypothalamus, where DRR1 expression is strongly induced by dexamethasone in hypothalamic neural-progenitor/stem cells derived from mouse embryos (Frahm et al. 2016, 2017), integrates inputs from many central and peripheral sources to maintain homeostasis of different organ systems and whole body metabolism (Denton et al. 1996).

Glucocorticoid receptor activation is able to increase the mRNA expression in almost all brain regions, except for few regions such as layer V from cortex or stratum radiatum in the hippocampus, where DRR1 mRNA expression is also not detected under baseline conditions. However, it is particularly intriguing that DRR1 expression is increased by glucocorticoids especially in white matter tracts, i.e., corpus callosum, anterior commissure. One possible explanation is that DRR1 mRNA could be strongly induced in oligodendrocytes, where DRR1 is lowly expressed under baseline conditions (Cahoy et al. 2008), and/or a consequence of an increase in corticosteroid-induced oligodendrogenesis (Chetty et al. 2014). In this line, glucocorticoid receptor expression is especially strong in white matter tracts in the adult mouse (Quinn et al. 2016). Another possibility is that DRR1 mRNA is transported to the terminal, as for example the actin-regulating protein Arc (Huang et al. 2007). Accordingly, stress-induced DRR1 protein expression is increased in membrane structures, but not in the nucleus. However, changes in the ratio of G-/F-actin in the cytosol could modulate serum response factor responsiveness (Olson and Nordheim 2010; Knöll 2011). Thus, stress might indirectly modulate gene transcription of immediate early genes and of actin cytoskeleton genes through DRR1 changes.

In conclusion, we provide further evidence that the actin-interacting protein DRR1 expression is induced by glucocorticoids, especially in membrane-rich structures and white matter tracts. As glucocorticoids-mediated mechanisms are prominent in the late phase response to stress (de Kloet et al. 2005), DRR1 induction could be involved in homeostasis restoration after stress and the storage of information in preparation for future use. However, stress disorders are more prevalent in women than in men; thus, further studies comparing male and female DRR1 expression and regulation by glucocorticoids should be addressed for translational relevance. Finally, these data might be a useful to understand DRR1-dependent physiology and to extend the knowledge on stress resilience mechanisms that could help to develop future therapeutic interventions.

## Electronic supplementary material

Below is the link to the electronic supplementary material.

Supplementary material 1 (DOCX 9746 KB)
