# Supplementary material for: Expression and glucocorticoid-dependent regulation of the stress-inducible protein DRR1 in the mouse adult brain
Source: Brain Struct Funct. 2018 Aug 18;223(9):4039–52. doi: 10.1007/s00429-018-1737-7 (PMC6267262; doi:10.1007/s00429-018-1737-7)
Supplement: Supplementary file 1 — Supplementary material 1 (DOCX 9746 KB) [file 429_2018_1737_MOESM1_ESM.docx]

**Figure S1**

**
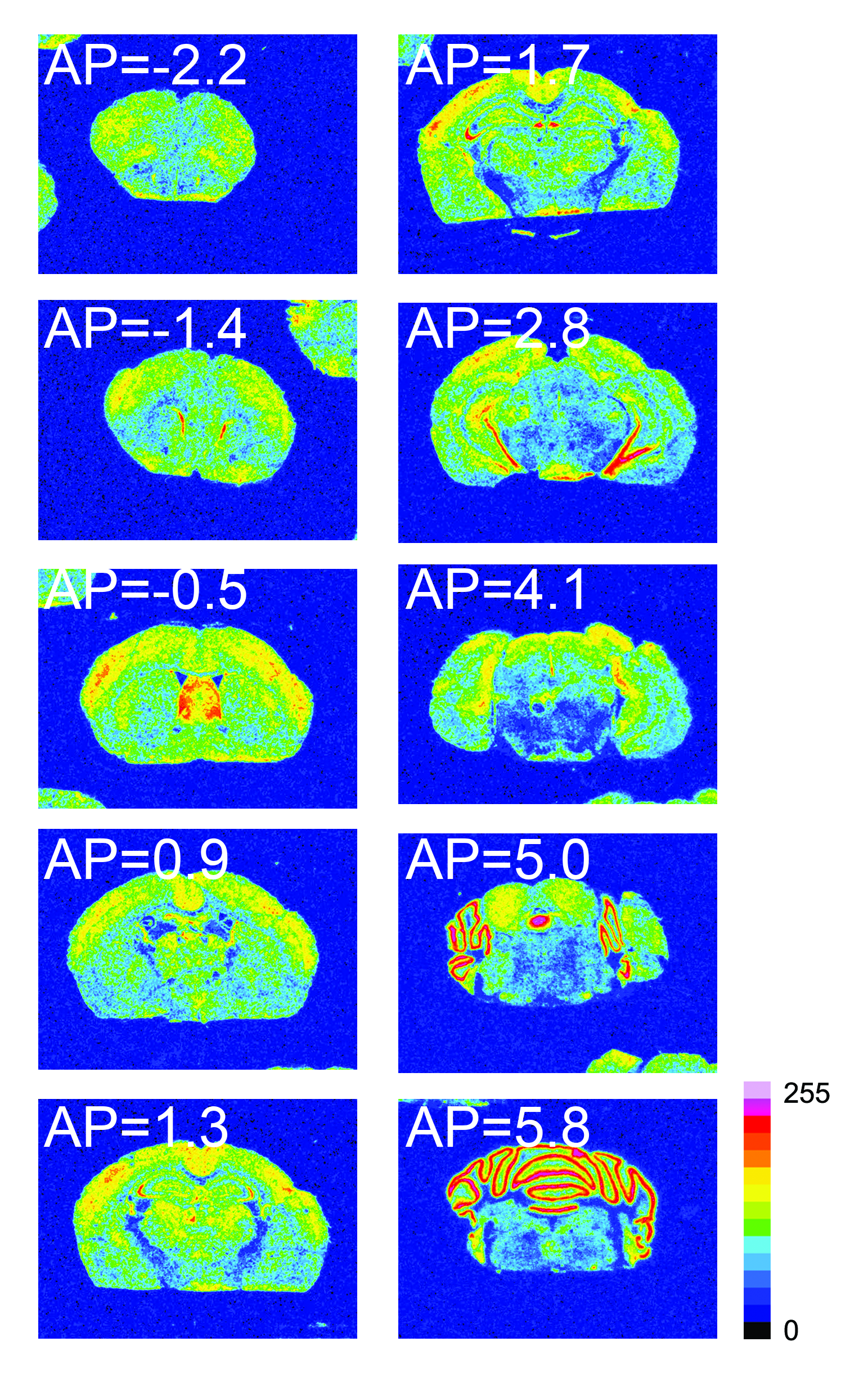
**

**Figure S1.** Representative *in situ* hybridization autoradiograph of [^35^S]-labelled DRR1 mRNA from coronal brain slices of one mouse. Original grey images were transformed to 16 colors range to emphasize differential mRNA expression levels throughout the adult mice brain. DRR1 mRNA expression was classified into four categories in table 1 following the color-scale of mRNA expression levels: red corresponds to +++ strong signal, yellow corresponds to ++ moderate signal, green corresponds to + weak signal and blue corresponds to – not detectable mRNA expression, as described in Table1.

**Figure S2

**

**Figure S2 cont**





Figure S2. High resolution photomicrographs showing the expression pattern of DRR1 mRNA revealed by silver grain staining under dark-field illumination. Pictures show mosaics of several photomicrographs at (a) AP: -0.3; (b) AP: 5.5; (c) AP: 1.8; (d) AP: 2.5 and (e) AP: 3.1 mm from bregma (Paxinos and Franklin 2008).

**Figure S3**

**
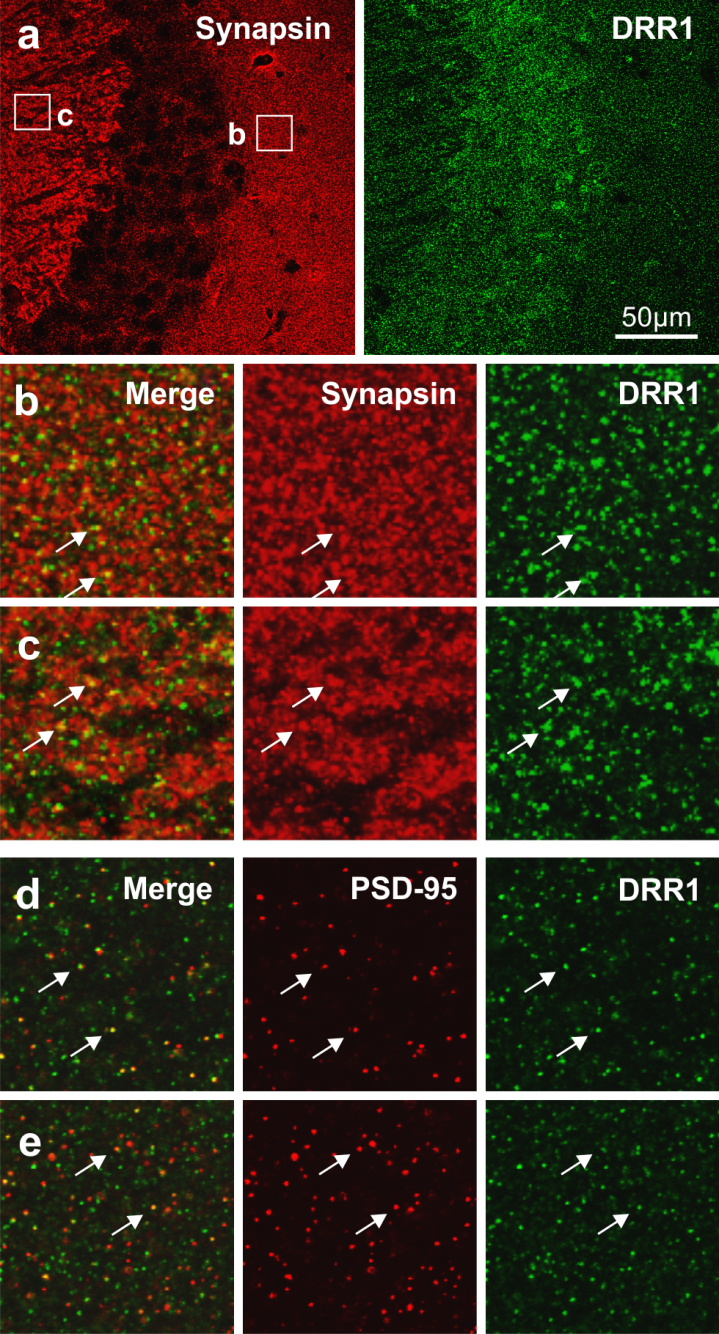
**

**Figure S3:** Synaptic localization of DRR1 protein. (a) DRR1 protein (green) can be found throughout the CA3 region within the cells of the pyramidal layer as well the stratum lucidum (SLu) and the stratum oriens (Or), whereas Synapsin (red) is located to the neuropil of SLu and Or. DRR1 is partly colocalized with Synapsin on both the Or (b) and SLu (c), as indicated (arrows). Colocalization of DRR1 and PSD-95 is shown (arrows) for SLu (d) and Or (e).

**Figure S4**

**Figure S4.** Corticosterone levels were measured 8h after dexamethasone (10mg/kg s.c), using a radioimmune assay. Dexamethasone treated mice showed reduced corticosterone levels compared to vehicle (n=16 mice/group). Bars show mean ± SEM. ***p < 0.0001 vs vehicle (t test).
